# The Medical Colleges of the City; Their Closing Exercises

**Published:** 1889-04

**Authors:** 


### THE MEDICAL COLLEGES OF THE CITY—THEIR CLOSING EXERCISES.

### ATLANTA MEDICAL COLLEGE.

THE closing exercises of the Atlanta Medical College were held at DeGive's Opera House, Monday evening, March 4th. The music dispensed by Wurm's orchestra was delightful, and added much to the pleasures of the occasion.

After prayer by Rev. A. F. Sherrill, D. D., the report of the Proctor, Dr. W. S. Kendrick, was read, showing that during the winter 117 students were enrolled from this and adjoining States; of which there were 65 in the first course and 52 in the second.

Forty-three names were presented for the degree of Doctor of Medicine, composed of the following: J. P. Bowdoin, P. E. Carr, J. T. Cobb, J. C. Embry, Geo. C. Erwin, A. T. Ford, Cicero Gibson, A. J. Gilbert, A. S. Gilbert, W. C. Hanson, H. F. Harris, J. W.

Hewell, P. M. Hodgson, R. L. Hollis, A. S. Howard, Paul Kendall, B. M. Kennon, M. B. McAfee, W. L. McBath, J. J. McEvoy, R. D. McLeod, L. B. McWhorter, H. L. Mobley, J. W. Neal, J. M. Neil, W. L. Patton, J. W. Peek, Paul E. Pennington, Y. L. Poole, D. E. Quillian, J. D. Robinson, J. L. Simpson, Jas. W. Smith, T. E. Stokes, F. W. Storey, G. W. Strickland, C. W. Taylor, Scott Thomson, W. W. Tison, W. J. Warren, G. W. Westbrook, Willis Winn, E. L. Wright.

Dr. H. H. Tucker, in his usual happy style, conferred the degrees, as President of the Board of Trustees. Rev. Dr. G. B. Strickler, the orator of the evening, followed in an admirable address on the subject of Evolution. Dr. J. P. Bowdoin, the Valedictorian of the class, did himself and the occasion justice in a concise, appropriaet speech. The honors were delivered by Dr. T. R. Kendall, of Macon, to the following gentlemen: Dr. H. F. Harris, first; Dr. W. C. Hanson, second, and Dr. J. W. Hewell, third. A large audience was present, and seemed to enjoy the evening very much, and by ten o'clock was dismissed by Rev. M. L. Underwood.

### SOUTHERN MEDICAL COLLEGE.

The Tenth Annual Commencement Exercises of the Southern Medical College were held at DeGive's Opera House March 2d. Exercises opened with prayer by Rev. H. K. Walker, of Marietta, Ga. The valedictorians, Dr. S. W. Jackson, of the Medical Department, and Dr. B. R. McBath, of the Dental, acquitted theselves with credit; both were eloquent and pathetic,

The annual oration, by Rev. H. K. Walker, setting forth the moral character of young physicians, was appreciated by both old and young of the profession. The past session has been a prosperous one, there being an increase not only in numbers but in ability as well. The degree of Doctor of Medicine was conferred upon the following 35 men, representing those who passed satisfactory examinations:

T. C. Avery, Ga.; J. T. Barnwell, Ga.; W. R. Belcher, Ala.; T. N. Bingham, Ga.; C. A. Blount, N. C.; R. L. Boynton, Ga.; W. C. Bryant, Ga.; W. W. Carmichæl, Ga.; E. H. Cook, Me.; W. H. Coston, Ala.; J. A. Craven, N. C.; D. M. Davis, Ala.; J. J. Foster, Ga.; W. T. Haygood, Ga., E. J. Hawkins, Ala.; J. L. Hilt, Ala.; W. A. Hitchcock, Ga.; W. E. Holtzclaw, S. C.; S. W. Jackson, Ala.; R. A. Justice, Ga.; A. P. Kemp, Ga.; W. M. Kemp, Ga.; J. R. Kinney, Ga.; W. C. Kiser, N. C.; B. B. Lancaster, N. C.; J. W. Nelms, Ga.; W. W. Redland, Ala.; B. D. Smith, Ga.; R. W. Trotter, Ga.; T. W. Tucker, Ga.; M. M. Warren, Miss., J. C. White, Ga.: W. M. Wilson, Ga.; J. H. Winchester, Ga.; W. T. Wise., Ala.

Two Gold Medals are awarded by the faculty to the two men passing the best examination in all branches, also each Professor gives a prize for the greatest proficiency in his branch. The following are the honor list:

First Honor, J. T. Barnwell, Ga.; Second Honor, W. T. Wise, Ala.; Obstetrics and Diseases of Women, J. C. White, Ga.; Practice Medicine, T. W. Tucker, Ga.; Materia Medica and Therapeutics, T. C. Avery, Ga.; Eye, Ear and Throat, W. A. Hitchcock, Ga.; Physiology, W. E. Holtzclaw, S. C.; Surgery, M. M. Warner, Miss.; Anatomy, B. D. Smith, Ga.; Chemistry, E. H. Cook, Me.

The Dental Department of the College, though only in its second year, has grown to considerable prominence.

### LIST OF GRADUATES.

J. A. Arbeely, M.D., Ga.; Aaron Branch, Ga.; O. H. Cantrell, Ga.; M. Z. Crist, Ky.; J. W. Daniel, La.; J. W. Duke, Ga.; C.

W. Forehand, Ga.; H. J. Garland, Ga.; R. L. Lane, Ala.; J. A. Link, Ga.; S. W. Lide, Ga.; B. R. McBath, Tenn.; L. B. Pitcher, Ga.; R. G. Ragan, Ala.; W. L. Sinclair, N. C.; H. B. Williamson, Ala.; S. M. Hyman, Ga.

---

### ITEMS.

WE received a pleasant call from Mr. N. E. Hulbert, who represents the Hoff's Malt department of the extensive business of Tarrant & Co., New York, and from him we learn that the genuine and only imported Johann Hoff's Extract of Malt is having an increased demand among the medical profession at the South. Mr. Hulbert will remain in Atlanta several days, during which time he intends calling on the physicians.

A CORRESPONDENT to the *Philadelphia Medical Times*, writes that one of his patients is affected with asthma, and can only live in comfort in the hills of Montana; while his wife is only free from rheumatism while in Southern California. Does any one know of a neutral ground, where these unfortunates may enjoy life and each other's society at the same time? Or would inability to come within 1000 miles of each other constitute legal grounds for a divorce?

A NEW MEDICAL COLLEGE.—We learn from authentic sources that the "Western North Carolina Medical College" will be established in this city at an early date. The leading spirits in the movement are Drs. S. W. Battle, F. T. Meriwether and J. A. Watson, and the college will be conducted upon the same rules governing instruction, etc., of other colleges of similar character. Application for a charter for the institution is now pending in the General Assembly.—*Asheville* (N. C.) *Citizen.*

THE annual meeting of the National Association of Railway

Surgeons will be held at St. Louis, Mo., on Thursday and Friday, May the 2d and 3d, 1889. The prospects are that this will be one among the largest gatherings of medical men ever assembled in this country. Dr. W. B. Outten, of St. Louis, is the Chairman of the Committee of Arrangements, and everything will be complete for the accommodation of the surgeons. Any information desired can be had by addressing the Secretary, C. B. Stemen, M. D., Fort Wayne, Ind.

SOME MEXICAN SUPERSTITIONS.—It is believed that the murderer who has slain his victim with sword or dagger will escape if the body falls upon its side or back; but if the body falls face downward, then the murderer surely soon will be captured and put to death. This belief is said to be so firmly rooted among the people of northeastern Mexico, that when a murdered man falls upon his face his slayer makes no effort to escape, and even sometimes voluntarily surrenders himself to justice.

If a bride, while dressing for her wedding, is pricked by a pin so that blood flows, great misfortune impends.

If two people think of the same thing at the same time, a soul is loosed from Purgatory.—Thomas A. Janvier, in *Scribner's Magazine* for March.

SEVEN WONDERFUL CURES.—In the Grub Street *Journal* of June 24th, 1736, appeared some lines on "Seven Wonderful Cures," a much advertised quack medicine of those days, which are sufficiently amusing to warrant reproduction:

One felt his sharp rheumatic pains no more,
A second saw much better than before;
Three cur'd of stone, a dire disease and sadder,
Who still, 'tis thought, have each a stone in bladder.
* * * * *
The last a little woman but great glutton,
Who at one meal eat two raw legs of mutton:
Nor wonder, since within her stomach lay
A wolf, that gap'd for victuals night and day;
But when he smelt the pill, he straight for shelter
Ran slap into her belly helter skelter.

—*Medical Classics.*

THE first triennial prize of two hundred and fifty dollars under the deed of trust of Mrs. Wm. F. Jenks, has been awarded by the Prize Committee of the College of Physicians of Philadelphia, to John Strahan, M. D., M. Ch., M. A. O. (Royal University, Ireland), 247 North Queen St., Belfast, Ireland, for the best essay on "The Diagnosis and Treatment of Extra Uterine Pregnancy."

The writers of the unsuccessful essays can have them returned to any address they may name, by sending it and the motto which distinguished the essay, to the Chairman of the Prize Committee, Ellwood Wilson, M. D., College of Physicians, Philadelphia.

The Trustees have made arrangements with Messrs. P. Blakister, Son & Co., 1012 Walnut St., Philadelphia, for the publication of the successful essay, which will also appear in the Transactions of the College for 1890.

Mr. Joseph Jefferson has been engaged for a number of years upon his autobiography, which will soon begin to appear in *The Century*. No more interesting record of a life upon the stage could be laid before the American public, and Mr. Jefferson's personality is perhaps more sympathetic to the people of this country than that of any actor we have had. He is the fourth in a generation of actors, and, with his children and grandchildren upon the stage, there are six generations of actors among the Jeffersons. The record which he has made of the early days of the American stage is said to be peculiarly interesting, especially the story of his travels as a boy in his father's company, when they would settle down for a season in a Western town and extemporize their own theater. The autobiography will begin in *The Century* during the coming autumn, and the installments will be illustrated with a portrait gallery of distinguished actors.

THE general state of the medical profession in Russia appears to be very unfortunate. Dr. C. Yaroshevski, in the *Russkaya Meditsina*, states that, though the number of doctors in proportion to the population is very much less than in other European countries, yet the destitution amongst them is alarming. Of late there have been numbers of suicides of medical men who were without the bare necessaries of life. The fees for medical attendance are very low. There are, he says, 18,000 doctors for a population of one hundred millions, or one medical man to every 6,500 persons. In Odessa 40 per cent. of the whole population, and 94 per cent. of the very poor, are stated to have died without having had medical attendance. A similar state of affairs exists at Kostrome. Dr. Yaroshevski attributes this deplorable condition of things to the ignorance of the Russian people, who prefer to consult soothsayers and magicians, to the monopoly enjoyed by the pharmacists, and to the large number of Feldshers who are allowed to practice. The Feldshers are generally men who have served in the Ambulance Corps or have been hospital attendants, and on the strength of this slight knowledge they are licensed to practice.—*British Medical Journal.*

THE Harvard Medical School has just received from Dr. D. W. Cheever the sum of $5000 to establish a scholarship to be known as the David Williams Cheever Scholarship.

A SURE SIGN.—"What an intellectual couple Mr. and Mrs. Cranque are!" "Intellectual? What makes you think so?" "Why, Mrs. Cranque cuts her hair short, and Mr. Cranque lets his grow long."—*Harper's Bazar.*

THE Pittsburg *Medical Review* thinks it well for some medical schools that they require as a condition of graduation, that the graduates shall not attempt to settle in Virginia, or if he does, that he shall never reveal the name of the medical school from whence he graduated.

A BUILDING site of 75 feet by 100 feet, with the privilege of an additional 25 feet, it is stated, has been secured on West Forty-third Street, near Fifth Avenue, for the new building of the New York Academy of Medicine.

"AND that is silver ore, is it?" said Mrs. Snaggs, as she examined a piece of curious looking mineral. "Yes, my dear," said her husband. "And how do they get the silver out?" "They smelt it." "Well, that's queer," after applying her nose to the ore. "I smelt it, too, but didn't get any silver."

THE *Westnik ssudebnoi mediciny i obschtschestwennoi gigieny*, hitherto published as a journal of forensic medicine and public hygiene, announces that it will hereafter be a journal of general medicine, and its title will be changed to *Westnik obschtschestwennoi gigieny, ssudebnoi i praktitscheskoi mediciny.*—*Ex.*

Dr. Virgil O. Hardon has removed to 38 North Forsyth St., where he has opened a Private Infirmary for Diseases of Women, fitted with all the most approved appliances for the treatment of this class of diseases. An institution of this kind has long been needed in Atlanta, and cannot fail to meet with success.

GLYCERINE as a remedy for constipation was for many years advertised as a secret remedy by a Holland doctor. The German Government, to aid him in filling his coffers, forbade its sale and published its analysis. As the profession ascertained its composition it began to employ it, and in slight cases of constipation find it of value.

MRS. HAYSEED—So young Wiggins is dead. I wonder what he died of?

Alonzo Hayseed (from college)—I heard it was pulmonary phthisis.

Mrs. H.—Land o'Goshen! and me thinkin' all the time the fellow had consumption.—*America.*

BY the will of the late Alexander Murray, of Montreal, the General Hospital of that city will receive the sum of $750,000. This is the largest gift hitherto made in Canada for any public charity, with the exception of the joint donation of Sir Donald A. Smith and Sir George Stephen, of a million dollars for the founding of the Royal Victoria Hospital, also of Montreal.

FOR WHAT ARE DOCTORS PAID?—An English judge has recently given a decision containing a great deal of common sense. A local surgeon sued the executor of a deceased farmer for an amount for consultations. The deceased could not take any medicine. The judge, in giving his decision, said: "Many people of a better class hold the idea that it is medicine doctors are paid for. It is for skill."

A BANGOR lady was very ill a few days ago, and a physician was called, who prescribed for her. The prescription was given to a servant girl, who was directed to take it to a drug store and have it filled. She went to the drug store, but, instead of carrying out her instructions, bought a postage stamp, placed it upon the prescription and dropped it into the post-office. In a short time it returned to the physician who gave it. It may be well to add that the lady did not die from the delay in receiving the medicine.

ACCCOUNTS RENDERED QUARTERLY.—There was a time when the services of physicians were not considered as an article of merchandise, with a fairly definite price, but rather as acts of benevolence and humanity, and then grateful patients signified their appreciation of these services by gifts in the nature of an honorarium. But this time has passed away, and now every medical man is compelled to keep accounts, and periodically to try to collect what he believes is due him by the unromantic method of sending out bills.—*Ex.*

A KENTUCKY paper says that the bill in that legislature, that provides that no doctor shall practise in any township without first depositing $100 to be used for charitable purposes, should be so amended as to require the patient, whose disease baffles the skill of the local doctor, to pay a fine of $500 every time he goes to a city to consult a specialist, the money to be used in filling the depleted State treasury.

A WISE JUDGE.—In a Berlin police court not long since a doctor was arraigned on a charge of assault. It seems that the doctor was called upon to prescribe for a boy about five years old with some slight indisposition. The little rascal screamed and kicked so viciously that the physician was unable to examine him, and after trying in vain to soothe him, the medical man, yielding to an impulse we all have felt, gave his patient something substantial to cry about. The child's mother, not relishing this part of the treatment, summoned the doctor before a magistrate. The worthy representative of Solomon decided that the doctor had acted for the best interests of his patient, and so acquitted him.—*Pittsburgh Med. Review.*

GOOD ADVICE.—A doctor in Liverpool has recently written to the press complaining that a prescription which he gave to a patient was being used by that patient to cure a great number of his friends. He told the man, who was a carpenter, that it was just as unfair to lend his prescription as it would be for the doctor to borrow the joiner's tools and lend them to his friends. He suggested that the proper way for the patient to do was to send these sick people to him. The moral he deduces is, that doctors should not give prescriptions to patients. If the doctor does not dispense, he should send the patient with the prescription, under cover, to a chemist, who should have instructions not to deliver a copy of it to the patient unless specially ordered to do so.—*Canada Med. Record.*

PECULIAR ASSAULT AND BATTERY.—A queer case was recently adjudicated by the correctional tribune of Lübeck. An old family physician—a man past sixty-five years of age—was arraigned for assault and battery upon a patient. It appeared from the evidence that the latter was a girl in her teens, whom the doctor had ordered to take certain medicines. On making his next visit he found that she had failed to do so, and without more ado he turned down the bed-clothes and spanked her soundly, *propria manu!* The tribunal sentenced him to nine months' imprisonment and a fine. It is likely that the former portion of the sentence will be remitted, as his many patients have petitioned the court to this effect.—*National Druggist.*

THE LOMB PRIZE ESSAY OF THE AMERICAN PUBLIC HEALTH ASSOCIATION.—The Association's Secretary, Dr. Irving A. Watson, of Concord, New Hampshire, has issued a circular setting forth its dissatisfaction with the way in which the New York *Herald* has dealt with it in the matter of the publication of the prize essay on "Practical Sanitary and Economic Cooking for Persons of Moderate and Small Means." It seems that the exclusive right of publication was given to the *Herald*, and that the Association's representatives understood that the essay was to be published entire. It now complains, not only that the *Herald* published only parts of the essay, with transpositions and violations of sequence that rendered the printed version comparatively worthless, but that it interpolated vulgar head-lines. We do not wonder that Dr. Watson and Mr. Lomb are indignant. Perhaps there is no redress for them in this instance, but their misfortune may serve the good purpose of impressing more and more upon those who have literary wares to purvey, that there should be some conformity between the character of matter to be published and and that of the proposed vehicle of its publication.—*New York Med. Journal.*

The following papers will be read before the coming meeting of the Medical Association of Alabama, at its next meeting in Mobile, April 10th to 14th, inclusive:

W. E. B. Davis, M. D., Birmingham, Electricity in Gynecology; Richard M. Fletcher, M. D., Madison, Eclampsia Gravidarum; Vivian P. Gaines, M. D., Mobile, Curability and Treatment of Pulmonary Phthisis; Thomas J. Johnson, M. D., Decatur, Fever: Its Etiology, and its Relation to Increased Temperature; Thomas J. Lee, M. D., Childersburg, Diagnosis and Treatment of Uterine Displacements; William M. Price, M. D , Florence, Cerebro-Spinal Meningitis; E. Powell Riggs, M. D.. Birmingham, Dysmenorrhœa: Its Causes and Treatment; E. Camp Wheeler, M. D., Cherokee, Dentition.

MEDICAL AND SURGICAL REGISTER OF THE UNITED STATES.—Messrs. R. L. Polk and Company, publishers of the *Medical and Surgical Register of the United States*, announce that they have now in preparation the second edition of the *Register*, which affords a convenient opportunity to the medical profession of the country to secure the best directory of the physicians of the United States that has ever been published, and with it much valuable information concerning medical matters, not given elsewhere in any single work. The publishers have offices in Detroit, Chicago, Baltimore, Atlanta, St. Paul, Minneapolis, Indianapolis, St. Louis, Toronto, and Portland, Oregon. Every physician is interested in making it correct and accurate, and should promptly give all the information that is desired of them.

AT the meeting of the trustees of the University of Pennsylvania, held last Tuesday, the following appointments were made: Dr. De Forest Willard, Clinical Professor of Orthopœdic Surgery; Dr. George A. Piersol, Professor of Histology and Embryology; and Dr. Samuel G. Dixon, Professor of Hygiene.

It is reported that the trustees have under serious consideration the advisability of adding in the near future a compulsory fourth year to the medical curriculum.

It is stated that Mr. Henry C. Lea has given $25,000 to the Laboratory of Hygiene of the University of Pennsylvania, on condition that an equal amount be obtained from other sources, and that a goodly proportion of this additional sum has been already raised.

The lecture upon hygiene and laboratory work next year will be obligatory upon the first year's students at the University.—*Ex.*

A CASE OF REINFECTION WITH SYPHILIS.—The case is recorded in the *Giornale delle Mal Ven*, of a woman, 46 years old, who had contracted syphilis when a child from a schoolfellow. The patient suffered very severely at the time, and had the characteristic eruptions, tibial pains, sore throat, and subsequent abortions. Ten years later she had well-marked tertiary manifestations, which gained for her the soubriquet of *fistolosa*, in her village. While she was still in the same deplorable condition she was reinfected by her husband, who had a chancre on the penis and was covered with a rosolar eruption. She came to the hospital, and was found to have a typical indurated chancre on the labium with corresponding glandular enlargement, and a confluent rose-colored papular eruption all over the body. She also complained of general malaise, and pain in the shoulders and knees. These symptoms promptly yielded to treatment, and there would not seem to be any reason to doubt the accuracy of the diagnosis in both instances. Indeed, that exceptions should exist in the protection afforded by a prior attack of syphilis is only what a study of other specific diseases would lead one to expect.—*Medical Press and Circular*, Feb. 6, 1889.

